# Measuring residual anti‐Xa activity of direct factor Xa inhibitors after reversal with andexanet alfa

**DOI:** 10.1111/ijlh.13591

**Published:** 2021-06-05

**Authors:** Matthieu Bourdin, Delphine Perrotin, Olivier Mathieu, Tristan Herve, François Depasse, Genmin Lu, Pamela B. Conley, Geneviève Contant

**Affiliations:** ^1^ Diagnostica Stago R&D Gennevilliers France; ^2^ Diagnostica Stago Clinical and Pharmaceutical Development Asnières‐sur‐Seine France; ^3^ Portola, a subsidiary of Alexion South San Francisco CA USA

**Keywords:** andexanet, apixaban, edoxaban, residual concentration measurement, reversion, rivaroxaban

## Abstract

**Introduction:**

Andexanet alfa (AnXa) was developed for anticoagulant effect reversal of direct factor Xa inhibitors (DXaI) (apixaban, rivaroxaban, edoxaban) in emergency situations. Regular anti‐Xa assays are not suitable to evaluate anti‐Xa activity after AnXa administration because of the high sample dilution resulting in the AnXa‐DXaI dissociation which gives inaccurately high DXaI measured concentrations. This study aimed at developing dedicated STA‐Liquid anti‐Xa test set‐ups for accurately measuring DXaI after reversal with AnXa.

**Methods:**

Modified anti‐Xa test set‐ups, with reduced sample dilution, were developed to overcome regular assays limitations and to improve measured accuracy with results comparable to Portola microplate reference method used in clinical studies. Both regular and optimized assays were used to measure DXaI concentration in AnXa‐containing samples. Quality controls, normal pooled plasma spiked with five DXaI and three AnXa concentrations, samples from DXaI‐treated patients spiked with AnXa and ex vivo healthy volunteers having received both DXaI and AnXa were used.

**Results:**

The lower limit of quantitation of optimized anti‐Xa assays was <10 ng/mL with CVs ≤10%. DXaI samples containing 300 ng/mL and 1 µmol/L AnXa resulted in DXaI residual concentrations of 29‐72 ng/mL depending on the DXaI (76%‐90% reversal), compared to 20‐28 ng/mL with reference method (92%‐94% reversal) and 135‐165 ng/mL with regular assays (about 50% reversal).

**Conclusion:**

Modified test set‐ups are automated alternative to reference method with improved precision and reproducibility. They can be run in all laboratories where regular anti‐Xa assays are performed using commercially available reagents.

## 1 INTRODUCTION

Oral direct inhibitors of factor Xa (DXaI such as apixaban, rivaroxaban, edoxaban) are increasingly used in several clinical indications. DXaI are effective therapeutic agents for the prevention and treatment of venous thromboembolism as well as stroke prevention in nonvalvular atrial fibrillation.^1^, ^2^ DXaI show more predictable pharmacokinetic and pharmacodynamic profiles than vitamin K antagonists. However, major bleeding events have been reported for patients taking DXaI. Depending on the drug, the therapeutic indication, and the dosage, the rate of major bleeding in patients can reach 3%‐5% patient–year.^3^, ^4^, ^5^, ^6^ Because of a large increase in prescription of DXaI, more patients require urgent reversal. A DXaI anticoagulant reversal agent can be useful for the management of serious bleeding or before urgent procedures.^7^

Andexanet alfa (AnXa) is a first‐in‐class recombinant, modified human factor Xa (FXa) molecule developed to reverse anticoagulation in patients taking DXaI or indirect FXa inhibitors.^8^, ^9^, ^10^ AnXa is the only specific reversal agent approved for anticoagulation reversal in patients presenting with a life‐threatening bleeding episode while treated with rivaroxaban or apixaban.^8^, ^9^, ^11^, ^12^, ^13^

Dedicated anti‐Xa chromogenic assays using specific calibrators and controls are used to measure direct and indirect FXa inhibitor levels in plasma. In emergency situations, such as uncontrolled bleeding, DXaI testing is useful to assess the presence of the drug and measure the plasma concentration.^14^ As an example, according to the Subcommittee on Control of Anticoagulation of the International Society on Thrombosis and Hemostasis (ISTH), reversal procedure should be considered in patients with serious and life‐threatening bleeding if the DXaI concentration measured before AnXa administration exceeds 50 ng/mL.^7^ However, the regular anti‐Xa assays do not allow an accurate measurement of the residual anti‐Xa activity after reversal with AnXa because of the high sample dilution, which can result in erroneously elevated anti‐FXa levels.^15^ In contrast, the microplate anti‐Xa chromogenic assays with specific test set‐up using minimal plasma sample dilution can be used to measure the residual anti‐Xa activity after reversal. The optimized microplate anti‐Xa assay has been used in AnXa clinical studies with inhibitor‐specific calibrators. Results are expressed in ng/mL for DXaI.^13^

The objectives of our work were to develop automated suggested test set‐ups with commercial calibrators and reagents for the measurement of residual DXaI concentration (apixaban, rivaroxaban, and edoxaban) after AnXa administration and then to evaluate the performances of these modified anti‐Xa test set‐ups in comparison with regular anti‐Xa test set‐ups and the microplate reference method used by Portola during drug development using patient samples.

## 2 MATERIAL AND METHODS

### 2.1 Material

Apixaban, rivaroxaban and edoxaban were provided by the respective manufacturer Bristol‐Myers Squibb, Bayer, and Daiichi‐Sankyo. Stock solutions were prepared by solubilizing DXaI at about 400 µg/ml into 100% dimethyl sulfoxide (DMSO).

AnXa stock solution was provided by Portola as formulated frozen solution at 234 µmol/L.

Frozen Normal Plasma Pool (NPP) and Pool Norm (commercial freeze‐dried NPP) were provided by Diagnostica Stago (Asnières sur Seine).

Leftover frozen citrated plasma samples were obtained from patients treated with apixaban (10 patients), rivaroxaban (10 patients), or edoxaban (7 patients). Patients did not oppose the use of their plasma for research. Frozen ex vivo samples from healthy volunteers (three treated with apixaban and two treated with rivaroxaban followed by AnXa administration) were provided by Portola.^13^, ^16^

Anti‐Xa reagents from the Coamatic Heparin kit (Chromogenix) were used for the reference microplate method with inhibitor‐specific calibrators. STA‐Liquid anti‐Xa were used for automated assays with STA‐Apixaban, STA‐Rivaroxaban, and STA‐Edoxaban Calibrators (STA‐Calibrator‐0; 1; 2; and 3) and Controls on STA‐R Evolution, STA‐R Max, or STA Compact Max instruments (Diagnostica Stago). STA‐Calibrator‐0 is a normal plasma pool free of AnXa and DXaIs.

### 2.2 Spiked samples

Spiked samples were prepared with frozen NPP as follows: DXaI stock solution was diluted with saline to obtain DXaI intermediate solution. NPP samples were then spiked with this solution to obtain the following DXaI concentrations (with ≤1% dilution of the plasma): 0, 50, 100, 300, and 500 ng/mL of apixaban (0 to 1.09 µmol/L), rivaroxaban (0 to 1.15 µmol/L), and edoxaban (0 to 0.91 µmol/L). Additional low DXaI concentrations were obtained by diluting the 50 ng/mL plasma sample with NPP.

DXaI‐spiked samples were either directly frozen or spiked with AnXa and then frozen at −80°C.

In vitro reversal with AnXa was performed as follows: DXaI‐spiked samples and DXaI‐treated patient samples were supplemented with AnXa stock solution to obtain 0, 1.0, and 2.3 µmol/L of AnXa (with ≤2% dilution of the plasma). The 1 ml samples spiked with AnXa were mixed and incubated in a water bath at 37°C for 5 minutes. Samples were mixed again after incubation and were frozen at −80°C until analysis.

Frozen samples were thawed in a water bath at 37°C for 5 minutes before use. All tests were performed within 4 hours after thawing.

### 2.3 Microplate reference method

The microplate reference anti‐Xa method was performed as described previously.^13^ Briefly, the assay was adapted from the commercial Coamatic Heparin kit with modifications to reduce the effect of sample dilution. The reaction mixture consisted of plasma (75 µl), bovine Factor Xa (25 µl, 1x stock solution), S2732 substrate (25 µl, 2x stock solution, supplemented with 25 µl assay buffer). Following incubation at room temperature for 5 minutes, the reaction was stopped by adding quenching buffer (50 µl). Microplate reference method performance study was done in triplicate with five aliquots of samples spiked with 500 ng/mL of apixaban or rivaroxaban and 2.3 µmol/L AnXa.

### 2.4 Regular and modified test set‐ups

Regular anti‐Xa assays on STA‐R Evolution or STA‐R Max instruments were used according to the manufacturer's recommendations and package inserts to measure DXaI concentration before reversal. Plasma samples were incubated with STA‐Liquid anti‐Xa substrate (150 µL) for 4 minutes at 37°C. The reaction was then triggered with prewarmed STA‐Liquid anti‐Xa Factor Xa (150 µl) at 37°C. The apixaban and rivaroxaban measuring ranges were 23‐500 ng/mL and 25‐500 ng/mL, respectively, with a 1:44 overall sample dilution (30 µl at 1:4 dilution). The edoxaban measuring range was 20 to 400 ng/mL with a 1:14 overall sample dilution (50 µl at 1:2 dilution).

Compared to the regular anti‐Xa assays, the modified test set‐ups used neat plasma samples and less reagents. In order to observe the sample dilution effect on DXaI residual activity,^15^ a series of conditions with an overall sample dilution from 1:2.6 to 1:12 was automatically realized by the STA‐R Evolution with rivaroxaban‐spiked samples using STA‐Liquid anti‐Xa reagent.

The remaining experiments were conducted using the 1:2.6 sample dilution, which was the lowest working dilution that could be achieved when using liquid reagents. Plasma samples (125 µl) were incubated with STA‐Liquid anti‐Xa substrate (100 µl) for 4 minutes at 37°C. The reaction was then triggered with prewarmed STA‐Liquid anti‐Xa Factor Xa (100 µl) at 37°C and Optical Density variation over time (OD/minutes) was measured at 405 nm between 1.33 and 2.50 minutes for rivaroxaban, 0.83 and 1.67 minutes for apixaban, and 0.50 and 3.00 minutes for edoxaban.

Taking into account lower DXaI concentration after reversal, dedicated quality control plasmas were prepared. Either STA‐Calibrator‐0, Pool Norm, or frozen NPP was used as diluents to dilute STA‐Calibrator‐1 for apixaban or rivaroxaban and STA‐Calibrator‐2 for edoxaban and thus prepare extemporaneously ≈ 30 and ≈ 60 ng/mL QC plasmas. Similarly, assay calibrations had to be adapted. STA‐Calibrator‐2 for apixaban or rivaroxaban and STA‐Calibrator‐3 for edoxaban were automatically diluted by the instrument. DXaI concentrations in the test samples were interpolated by the instrument from the respective 2nd‐order polynomial calibration curves from 0 to 70 ng/mL depending on the initial calibrator titer. For concentrations between 70 and 130 ng/mL, residual DXaI concentrations were extrapolated from the raw data (OD/minutes) and the respective assay calibration curves allowing a measuring range from 10 (see LoQ determination below) to 130 ng/mL.

### 2.5 Results and statistical analysis

Reagents lots, replicates, instruments, and samples used in this work are detailed in Table 1. Three different reagent lots (STA‐Liquid anti‐Xa and STA‐Calibrators) and two different STA‐R Evolution, STA‐R Max, or STA Compact Max instruments were used. The reversal effect of AnXa was expressed either as DXaI plasma concentration in ng/mL or in percent reversal versus the initial DXaI plasma sample concentration.

**TABLE 1:** Evaluation of regular and modified test set‐ups with citrated plasma samples

ITEM	Test set‐ups	Anti‐Xa Reagent lots	Citrated plasma samples	Replicates	Instruments	Calculation mode^a^
% Reversal on Spiked Plasmas	Regular	1	Spiked AnXa‐DXaI	3	1	% Reversal
	Modified	3	Spiked AnXa‐DXaI	24	2	
Sample Dilution Effect	Regular	1	Spiked AnXa‐DXaI	3	1	Residual ng/mL
Limit of Detection (LoD)	Modified	1	Spiked DXaI	40	2	SD ng/mL
Lower Limit of Quantification (LoQ)	Modified	1	Spiked DXaI	40	2	% *vs* Reg. Assays
Within‐run	Modified	1	30 & 60 ng/mL QC	21	1	% CV
Day‐to‐day	Modified	3	30 & 60 ng/mL QC Spiked AnXa‐DXaI	24	2	
Diluent for Calibration	Modified	1	Spiked AnXa‐DXaI	3	1	% Reversal
Patient Samples Spiked with AnXa	Modified	1	10 Apixaban 10 Rivaroxaban 7 Edoxaban	2	1	% Reversal
Ex vivo Samples	Modified	1	3 Apixaban 2 Rivaroxaban	3	1	Residual ng/mL

^a^ All the performances were calculated using Excel sheets

Repeated measurements were used to establish the analytical performances of the modified anti‐Xa test set‐ups (Table 1). Limit of Detection (LoD) was determined based on method standard deviation (SD) obtained from samples with a relevant low concentration of DXaI (from 0 to 30 ng/mL). Lower Limit of Quantitation (LoQ) was determined in comparison to regular anti‐Xa assays with DXaI‐spiked samples (from 5 to 35 ng/mL) without AnXa and with a maximum acceptable bias of 10 ng/mL taking into account the precision and the accuracy of the method. Performances evaluation (precision, LoD, and LoQ) was estimated according to in vitro CLSI guidelines ^17^, ^18^ as presented in Table 1. Coefficient of variation (% CV) was used to compare modified anti‐Xa test set‐ups and microplate reference method.

## 3 RESULTS

### 3.1 Reversal measured with regular anti‐XA assays on spiked plasma

With regular anti‐Xa assays and spiked AnXa‐DXaI samples, the measured reversal varied from 49% to 56% with apixaban, from 47% to 62% with rivaroxaban, and from 57% to 73% with edoxaban. As an example, spiked samples containing 300 ng/mL and 1.0 µmol/L AnXa resulted in 144 ng/mL of apixaban (53% reversal), 165 ng/mL of rivaroxaban (48% reversal) and 135 ng/mL of edoxaban (57% reversal) (Table 2).

**TABLE 2:** DXaI reversal by AnXa measured in samples spiked with AnXa and DXaI with A. modified test set‐ups compared to microplate reference method; B. regular anti‐Xa assays

A. Samples Spiked AnXa‐DXaI	DXaI ng/mL	Modified Anti‐Xa test set‐ups			Reference Anti‐Xa method		
		Apixaban ng/mL (% reversal)	Rivaroxaban ng/mL (% reversal)	Edoxaban ng/mL (% reversal)	Apixaban ng/mL (% reversal)	Rivaroxaban ng/mL (% reversal)	Edoxaban ng/mL (% reversal)
1.0 µmol/L	50	6 (89%)	3 (94%)	7 (86%)	3 (95%)	3 (94%)	4 (95%)
1.0 µmol/L	300	72 (76%)	29 (90%)	47 (84%)	28 (92%)	20 (94%)	20 (94%)
2.3 µmol/L	50	3 (95%)	2 (96%)	4 (92%)	2 (96%)	1 (98%)	2 (98)
2.3 µmol/L	300	29 (91%)	8 (97%)	19 (94%)	7 (98%)	8 (98%)	9 (98%)
2.3 µmol/L	500	50 (90%)	18 (96%)	34 (93%)	16 (97%)	12 (98%)	14 (98%)

B. Samples Spiked AnXa‐DXaI	DXaI ng/mL	Regular Anti‐Xa Assays		
		Apixaban ng/mL (% reversal)	Rivaroxaban ng/mL (% reversal)	Edoxaban ng/mL (% reversal)
1.0 µmol/L	300	144 (53%)	165 (48%)	135 (57%)
2.3 µmol/L	500	ND	192 (62%)	134 (73%)

Abbreviations: ND, Not determined.

### 3.2 Sample dilution effect

**FIGURE 1:**
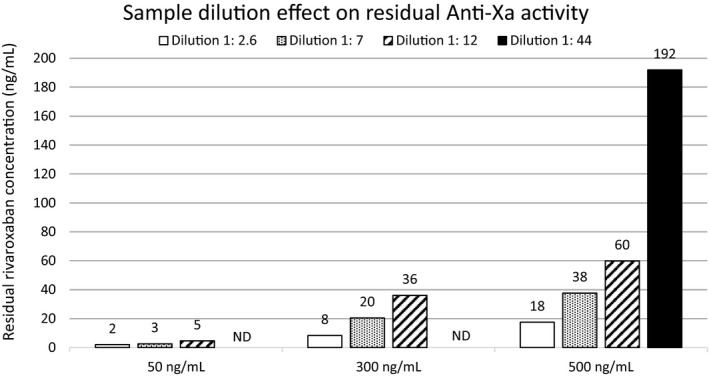
Sample dilution effect on residual anti‐Xa activity based on ng/mL. The results are compared following different dilutions with rivaroxaban (50 to 500 ng/mL)‐spiked plasma samples and AnXa concentration of 2.3 µmol/L. The residual concentrations are expressed in ng/mL for each bar which corresponds to a sample and its dilution

With the modified test set‐up, results obtained with rivaroxaban‐spiked samples varied according to the dilution as shown in Figure 1. The measured residual DXaI concentration decreases with decreasing dilutions. A maximum reversal is observed with the 1:2.6 sample dilution.

### 3.3 Technical performances

Based on the standard deviation analysis, the highest LoD was 6 ng/mL obtained with the modified rivaroxaban test set‐up. With a maximum bias of 10 ng/mL compared to STA‐Liquid anti‐Xa regular assays, the LoQ was estimated to be less than 10 ng/mL for all DXaI. Modified apixaban test set‐up bias was found above 10 ng/mL for concentrations higher than 30 ng/mL.

The maximum CVs obtained for the precision (within‐run and inter‐assays) for all the QC levels were less than 8% for all DXaI. For the AnXa‐DXaI‐spiked samples, the maximum inter‐assay CV was less than 10%. The calibration curves for rivaroxaban, apixaban, and edoxaban assays showed a good correlation coefficient with *R* values ≥0.995 during the inter‐assay measurements (data not shown). Compared to the modified test set‐ups, CVs of triplicates obtained with the microplate reference method ranged from 2% to 18% for apixaban and from 6% to 30% for rivaroxaban.

The mean percent reversal with STA‐Calibrator‐0 was 91% compared to 91% obtained with Pool Norm and 94% with the frozen NPP, indicating minimal matrix effect with different diluents.

### 3.4 Reversal measured with modified test set‐ups on spiked samples

With modified test set‐ups and spiked AnXa‐DXaI samples, the measured reversal varied from 76% for apixaban to 97% for rivaroxaban (Table 2). Spiked samples containing 300 ng/mL and 1.0 µmol/L AnXa resulted in 72 ng/mL of apixaban (76% reversal), 29 ng/mL of rivaroxaban (90% reversal) and 47 ng/mL of edoxaban (84% reversal).

### 3.5 Patient samples spiked with AnXa

Samples obtained from DXaI‐treated patients and spiked with AnXa were tested with the modified test set‐ups (Table 3). After reversal with 1.0 µmol/L AnXa, samples with initial DXaI concentrations from 119 to 290 ng/mL were reduced to 23 to 65 ng/mL for apixaban (78%‐83% reversal), 7 to 19 ng/mL for rivaroxaban (93%‐96% reversal) and 21 to 38 ng/mL for edoxaban (84%‐87% reversal). With regular assays, same samples gave a percentage of reversal below 53%.

**TABLE 3:** DXaI reversal measured with regular assay and modified test set‐ups in patient samples with concentrations from 119 to 290 ng/mL and spiked with 1 µmol/L of AnXa

Test set‐ups	Apixaban 10 patients ng/mL (% reversal)	Rivaroxaban 10 patients ng/mL (% reversal)	Edoxaban 7 patients ng/mL (% reversal)
Regular assays	72‐157 (46%‐53%)	87‐148 (34%‐50%)	68‐243 (16%‐43%)
Modified test set‐ups	23‐65 (78%‐83%)	7‐19 (93%‐96%)	21‐38 (84%‐87%)

### 3.6 Ex vivo samples

**FIGURE 2:**
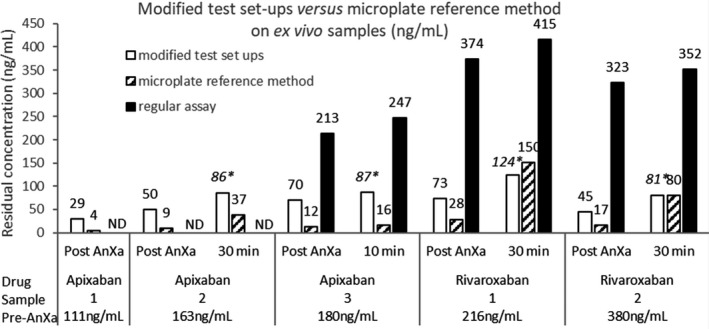
Ex vivo determination of residual DXaI concentration in five healthy volunteers samples treated by apixaban or rivaroxaban and after AnXa infusion. For apixaban‐treated subjects, the samples were take from the 420 mg bolus cohort with no follow‐on infusion ^16^ where rivaroxaban‐treated subjects were take from the 720 mg bolus +4 mg/min infusion cohort.^13^ Results are displayed for plasma samples drawn immediately after AnXa infusion (1 sample) and 10 (1 sample) or 30 min (3 samples) after AnXa infusion. The residual concentrations are expressed in ng/mL for each bar

Modified anti‐Xa set‐ups showed dramatic improvement in measuring AnXa reversal activity, but with a mean 18% and median 23% underestimation of percent reversal (ie, higher anti‐Xa activity) compared to the microplate reference method (Figure 2).

## 4 DISCUSSION

Andexanet alfa (AnXa) was developed for DXaI (apixaban, rivaroxaban, and edoxaban) reversal in emergency situations. It was recently shown that regular anti‐Xa assays are not suitable to evaluate anti‐Xa activity after AnXa administration.^15^

In the current study, we developed automated suggested test set‐ups suitable for the measurement of residual DXaI concentration of rivaroxaban, apixaban, and edoxaban after AnXa administration. We further evaluated their performances in comparison with regular anti‐Xa assays and microplate reference method with patient's samples. Thus, modified anti‐Xa test set‐ups were able to measure low residual DXaI concentrations in the presence of AnXa with an overall decreased sample dilution.^15^

Median DXaI concentrations reported in the literature before AnXa administration were 277 ng/mL for rivaroxaban, 150 ng/mL for apixaban, and 130 ng/mL for edoxaban.^9^, ^11^, ^13^ Because AnXa binds to DXaI with a 1:1 stoichiometry, a molar excess [AnXa: DXaI] would be needed to completely reverse anticoagulation, for example, about [1.2:1.0] for apixaban, [1.0:1.0] for rivaroxaban and [1.4:1.0] for edoxaban.^11^, ^12^, ^19^ We choose two concentrations of AnXa (1.0 and 2.3 µmol/L) in the spiking experiment to allow reversal of concentrations up to 500 ng/mL for the three DXaI. However, an incomplete reversal was observed in the excess amount of AnXa (2.3 µmol/L) using the regular anti‐Xa assays (Table 2).

Different test set‐ups were evaluated in rivaroxaban‐spiked samples in the presence of AnXa. We observed that dilution of the plasma sample had a major impact on results with an improved reversal measurement by decreasing the overall sample dilution in the reaction mixture. The optimal sample dilution among those tested was 1:2.6 in the modified test set‐ups. Three plasma sources, namely STA‐Calibrator‐0, Pool Norm, and in‐house frozen NPP were used as diluent with similar results, allowing to choose the most appropriate one in laboratory routine.

The inter‐assay precision was acceptable with CVs ≤10% for all DXaI.^20^ The lower limit of quantitation (< 10 ng/mL) of optimized anti‐Xa test set‐up was in line with clinical need. Indeed, reversal is indicated in patients with bleeding when DXaI concentration is above 50 ng/mL and 30 ng/mL. is the maximum recommended concentration for some invasive procedures.^7^

With DXaI‐AnXa‐spiked samples, the percent reversal measured with modified test set‐ups was much higher than the one with regular assays and close to the microplate reference method. This observation could be explained by the high sample dilution with regular assays (eg, 1:44 for rivaroxaban and apixaban and 1:14 for edoxaban) which causes dissociation between the inhibitor and AnXa.^15^ For these reasons, we confirmed that regular assays are not suitable for accurate measurement of anti‐Xa activity after AnXa administration.

Regarding the comparison between modified set‐up microplate and reference method, there are several sources of variability beyond sample dilutions (1:2.6 vs. 1:2) that might affect the anti‐Xa results, notably the nature and the concentration of the substrate and factor Xa and the measuring mode (kinetics vs end‐point). However, the modified STA‐Liquid anti‐Xa assay set‐ups were highly reproducible (much lower CVs than the reference method), allowing the best compromise between reversal measurement and assay analytical performance. In addition, it can be easily adapted on STA‐R Evolution, STA‐R Max, and STA Compact Max.

As observed with spiked samples, the residual DXaI concentration after AnXa reversal measured with microplate reference method which is manual was hampered by the high CVs. The modified test set‐ups were deemed acceptable on an analytical point of view due to better reproducibility with automated platforms. The clinical relevance of the percent reversal underestimation remains to be documented for patient management.

Although regular anti‐Xa assays can accurately measure DXaI concentrations before AnXa administration, they are not suitable for AnXa‐containing samples post‐AnXa treatment as demonstrated in this study and previous report.^15^ Therefore, it is important that the clinical laboratory is informed of the clinical context of the request and that clinicians are aware that they must inform the clinical laboratory of the request context (ie, whether the samples contain AnXa).

Our work has however some limitations. Rivaroxaban was considered as representative of DXaI for determination of the most appropriate sample dilution. As results show a different behavior of apixaban in terms of test performances, additional experiments should be carried out to verify the optimal sample dilution with this drug. The limited performance of apixaban test set‐up might be due to the higher bias observed with this molecule at concentrations above 30 ng/mL. Further experiments are needed to confirm these results. Moreover, lower sample dilutions could not be assessed due to the test set‐up automation constraints.

Statistical analysis of technical performances was limited by the small number of replicates compared to guidelines for in vitro testing.^16^, ^17^ Nevertheless, this allowed the first characterization of the performance of the modified anti‐Xa methods using commercial reagents on automated analyzers.

## 5 CONCLUSION

To the best of our knowledge, this is the first work on automated and dedicated coagulation test set‐ups for rivaroxaban, apixaban, and edoxaban anti‐Xa measurement after AnXa administration. We demonstrated that DXaI patient samples reversed with AnXa can be tested with these dedicated automated test set‐ups on STA‐R Evolution, STA‐R Max, or STA Compact Max using commercial Stago reagents.

## CONFLICTS OF INTEREST

The authors are full‐time employees of Diagnostica Stago, or Portola, a subsidiary of Alexion. Diagnostica Stago conducted the work independently from Alexion Pharmaceuticals except for the supply of andexanet alfa and patient samples.

## AUTHOR CONTRIBUTIONS

Conceptualization, methodology, formal analysis and writing MB, DP, OM, GC, TH and FD; investigation, DP and MB; resources, GL and PC; review and editing, GL and PC; supervision FD All authors have read and agreed to the published version of the manuscript.

## Data Availability

Alexion will consider requests for disclosure of clinical study participant‐level data provided that participant privacy is assured through methods like data de‐identification, pseudonymization, or anonymization (as required by applicable law), and if such disclosure was included in the relevant study informed consent form or similar documentation. Qualified academic investigators may request participant‐level clinical data and supporting documents (statistical analysis plan and protocol) pertaining to Alexion‐sponsored studies. Further details regarding data availability and instructions for requesting information are available in the Alexion Clinical Trials Disclosure and Transparency Policy at http://alexion.com/research‐development. Link to Data Request Form (https://alexion.com/contact‐alexion/medical‐information).
